# Genetic Approaches Using Zebrafish to Study the Microbiota–Gut–Brain Axis in Neurological Disorders

**DOI:** 10.3390/cells10030566

**Published:** 2021-03-05

**Authors:** Jae-Geun Lee, Hyun-Ju Cho, Yun-Mi Jeong, Jeong-Soo Lee

**Affiliations:** 1 Disease Target Structure Research Center, KRIBB, 125 Gwahak-ro, Yuseong-gu, Daejeon 34141, Korea; jglee89@kribb.re.kr (J.-G.L.); alleles@kribb.re.kr (H.-J.C.); angdoym@kribb.re.kr (Y.-M.J.); 2 KRIBB School, University of Science and Technology, 125 Gwahak-ro, Yuseong-gu, Daejeon 34141, Korea; 3 Dementia DTC R&D Convergence Program, KIST, Hwarang-ro 14 gil 5, Seongbuk-gu, Seoul 02792, Korea

**Keywords:** microbiota, gut–brain axis, zebrafish, genetic approach, in vivo imaging, gnotobiotic

## Abstract

The microbiota–gut–brain axis (MGBA) is a bidirectional signaling pathway mediating the interaction of the microbiota, the intestine, and the central nervous system. While the MGBA plays a pivotal role in normal development and physiology of the nervous and gastrointestinal system of the host, its dysfunction has been strongly implicated in neurological disorders, where intestinal dysbiosis and derived metabolites cause barrier permeability defects and elicit local inflammation of the gastrointestinal tract, concomitant with increased pro-inflammatory cytokines, mobilization and infiltration of immune cells into the brain, and the dysregulated activation of the vagus nerve, culminating in neuroinflammation and neuronal dysfunction of the brain and behavioral abnormalities. In this topical review, we summarize recent findings in human and animal models regarding the roles of the MGBA in physiological and neuropathological conditions, and discuss the molecular, genetic, and neurobehavioral characteristics of zebrafish as an animal model to study the MGBA. The exploitation of zebrafish as an amenable genetic model combined with in vivo imaging capabilities and gnotobiotic approaches at the whole organism level may reveal novel mechanistic insights into microbiota–gut–brain interactions, especially in the context of neurological disorders such as autism spectrum disorder and Alzheimer’s disease.

## 1. Introduction 

The microbiota–gut–brain axis (MGBA) is a bidirectional signaling cascade in which efferent signaling pathways originating from the central nervous system (CNS) regulate the activities of the intestine and the microbiota, while afferent signaling originating from the microbiota and the intestines affects the development and the function of the CNS [1]. The MGBA mainly consists of gut microbiota residing in the intestinal lumen, intestinal cells including enterocytes, enteroendocrine cells (EECs), goblet cells, and neurons and glia in the CNS. Gut microbiota have been shown to be required for normal CNS homeostasis. For example, germ-free (GF) mice have been reported to display hypermyelination in the prefrontal cortex [2] and to have defective microglial maturation and functions [3]. The actions of the MGBA are known to be mediated by metabolites and cytokines that are generated by members of the gut microbiota or released from immune cells and intestinal cells activated by them, or by the streamlined direct connections between the brainstem and intestines via the vagus nerve [4]. Imbalances of the gut microbiota, referred to as dysbiosis, and any associated malfunctions of the MGBA have been implicated in a variety of neurodevelopmental, neuropsychological, and neurodegenerative diseases. These dysbiotic malfunctions have been closely associated with aberrant systemic inflammatory responses and have been shown to culminate in the brain defects that lead to behavioral defects and neuronal dysfunctions [5]. Thus, a more detailed understanding of the underlying mechanisms and physiological roles of the MGBA in the etiopathology in diseases will help to design novel therapeutics based on modulating MGBA activities. For these purposes, the zebrafish has emerged as an excellent animal model system to address the host–microbe interactions for both normal physiology and pathogenesis in vivo. In this topical review, we summarize recent human and other animal model findings regarding the MGBA and discuss the characteristics and utility of using zebrafish as an animal model to study the MGBA. 

## 2. MGBA Pathways 

**Figure 1:**
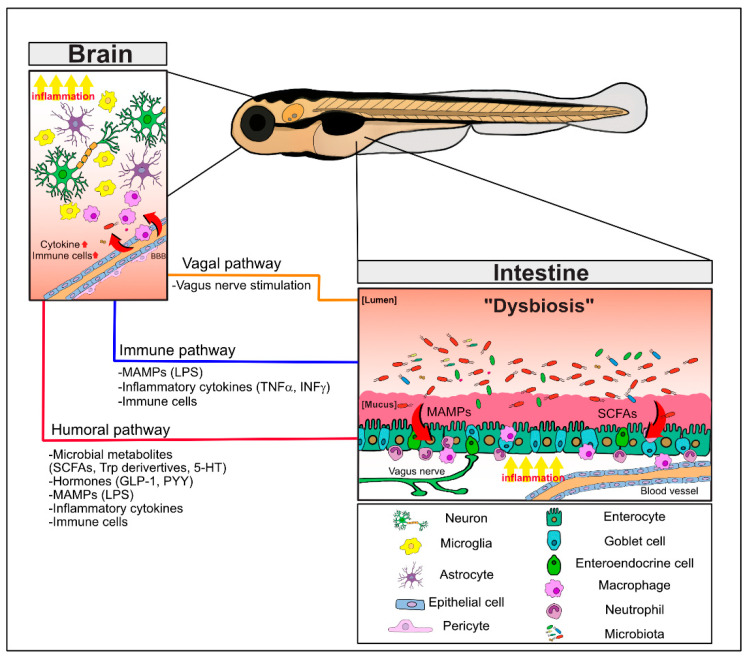
The bidirectional pathways of the microbiota–gut–brain axis (MGBA) involving the microbiota, the intestine, and the brain. Intestinal dysbiosis and derived metabolites in neuropathological conditions induce the barrier permeability defects and local inflammation with increased pro-inflammatory cytokines, MAMPs (e.g., LPS), and activation of immune cells in the gastrointestinal tract. These signaling mediators as well as microbial metabolites (e.g., SCFAs and Trp derivatives) and hormones (e.g., GLP-1, PYY) can distantly affect the brain function via the humoral pathway. In addition, dysregulated regulation of the vagus nerve can also directly modulate the brain function. Together, these MGBA pathways culminate in regulating neuroinflammation and neuronal defects of the brain and behavioral abnormalities. Refer to the text for details. 5-HT; 5-hydroxyltryptamine; LPS, lipopolysaccharides; MAMPs, microbe-associated molecular patterns; SCFAs, short chain fatty acids; Trp; tryptophan.

In a pioneering study, GF mice were shown to display exaggerated stress responses and enhanced stress hormone levels that were reversed by the colonization of beneficial bacteria or commensal microbiota, indicating that the gut bacteria play a critical role in the regulation of brain function [6]. Subsequent studies showed that GF mice exhibited hypermotor activity and reduced anxiety, concomitant with changes in gene expression profiles that are important for brain development, and with changes in essential neurotransmitters, specifically in the striatum [7]. The permeability of both the gastrointestinal barrier and the blood–brain barrier (BBB), separating luminal contents from the intestines or blood vessel contents from the brain, respectively, should be tightly regulated by the host to maintain their functional integrities, as the unchecked translocation of bacterial components and metabolites could elicit detrimental inflammation in both the intestine and the brain. Pro-inflammatory cytokines, such as tumor necrosis factor (TNF) and interferon γ (IFNγ), have been shown to regulate the functions of tight junctions [8,9,10]. The MGBA pathways and important signaling mediators are summarized in Figure 1.

### 2.1. Regulation of Brain and Intestinal Permeability by the Microbiota 

In GF mice, their intestinal barriers exhibited decreased permeability, with increased expression of several tight junction proteins and immature structural features, which were all reversed after associations with human commensal microbiota [11]. In addition, the composition of gut bacteria can directly affect gut permeability by determining its mucus layer properties [12], and pathogens such as *Bacteroides fragilis* and *Vibrio cholerae* or probiotic strains such as *Lactobacillus plantarum* can change intestinal permeability by regulating tight junction proteins [13], all indicating critical roles for gut microbiota in the regulation of gut permeability. As a result, the changes in intestinal permeability affected by infections or by exposure to dysbiotic products such as bacterial toxins and metabolites can result in low-level chronic and systemic inflammation, eventually developing into a variety of diseases [10,14].

The BBB in several brain areas is also regulated by the gut microbiota. However, in contrast to the intestinal barrier, it became more permeable in GF mice, with reduced expressions of endothelial tight junction proteins, and these were rescued after associating with the commensal microbiota [15]. Exposure to short chain fatty acid (SCFA)-producing bacteria or to butyrate treatment also reversed BBB permeability defects via the upregulation of tight junction proteins, indicating the crucial involvement of bacterial SCFAs in BBB permeability [15] as well as gut permeability [16]. 

### 2.2. Neuronal Communication via the Vagus Nerve

Both the expression of gamma-aminobutyric acid (GABA) receptors in the brain and the improvements in anxiety and depression behaviors regulated by probiotic lactic acid bacteria in mice have been shown to be mediated by the vagus nerve; this has been confirmed by vagotomy where the vagus nerve that connects the brain and the intestine is surgically disconnected [17]. In addition, a subset of intestinal EECs, termed neuropods, has been identified as forming physical synapses with the vagus nerve as part of a neuroepithelial circuit that can sense intestinal signals and convey that information directly to the brain [18]. A neuronal circuit via the vagal nerve has also been described in zebrafish (see below). 

### 2.3. Immune Cell Infiltration into the Brain and Inflammatory Cytokines

Several types of innate immune cells are found in the brain, including residential microglia, perivascular macrophages, and infiltrating macrophages. The infiltrating macrophages of the brain are derived from peripheral bone marrow pro-inflammatory monocytes that transmigrate across the BBB and differentiate into activated macrophages [19]. Neuroinflammation can provoke this type of infiltration, the hallmarks of which are activated microglia as well as the increased expressions of pro-inflammatory cytokines and chemokines such as interleukin-1β (IL-1β), IL-6, IL-17, TNFα, and monocyte chemoattractant protein 1 (MCP1, also called CCL2) in the inflamed brain [20,21]. The selective depletion of infiltrating monocytes worsened the Aβ load in the Alzheimer’s disease (AD) brain [22] and infiltrating macrophages were shown to have a higher phagocytic capacity against toxic molecules (i.e., Aβ) in AD compared to residential microglia [20,21], indicating that monocyte infiltration plays a critical role in Aβ clearance. Neutrophils have also been shown to infiltrate into the brain using mouse AD models, and were attracted to amyloid plaques in an LFA-1 integrin-dependent manner [23,24]. Neutrophil depletion, or LFA-1 blockade, was reported to reduce the neuropathological phenotypes and behavioral deficits in AD mouse models [24]. Furthermore, adaptive immune cells (e.g., clonally expanded CD8^+^ T cells) have been found in the cerebrospinal fluid (CSF), perivascular regions, and the parenchyma of AD brains [25,26], although any functional significance and detailed infiltration routes remain to be elucidated. 

The neuroinflammation that promotes the infiltration of peripheral immune cells into the brain is likely associated with MGBA function because systemic inflammation elicited by acute infections or by gut dysbiosis, such as inflammatory bowel disease (IBD), has been shown to be strongly associated with neuroinflammatory responses [27]. During dysbiosis, changes in beneficial or pathogenic bacteria and their metabolites (e.g., reduction of *Faecalibacterium prausnitzii* and its metabolite butyrate) may affect the integrity of intestinal barriers by regulating tight junction protein expressions [28,29], eventually resulting in the mobilization of activated immune cells in the intestine and the increased expression of inflammatory cytokines [14]. Infiltration of peripheral immune cells into the brain may be further facilitated by systemic inflammation because BBB permeability is also compromised as a result [10]. Consistent with this process, gut dysbiosis was closely associated with the infiltration of pro-inflammatory T helper 1 (Th1) cells into mouse brain using the 5XFAD model and with microglia differentiating into the pro-inflammatory M1 type [30].

The infiltration of immune cells into the brain due to intestinal inflammation has also been observed using a non-mammalian AD model. In a *Drosophila* model overexpressing amyloid β42 (Aβ42), a non-lethal enterobacterial infection promoted the infiltration of hemocytes (invertebrate phagocytic immune cells) and induced neurodegeneration via the TNF/JNK pathway due to enhanced oxidative stress [31].

### 2.4. MGBA Metabolites: SCFAs and Tryptophan Derivatives, Including Serotonin

Microbial metabolites have been proposed to be the mediators that link microbiota changes to host metabolism in both normal physiology and disease [32]. The effects of these metabolites on the CNS are via the activation of nerves innervating the intestine, activation/mobilization of immune cells residing in the intestine, or by activating the release of molecules (i.e., endocrine peptides and cytokines) via humoral pathways [33]. The SCFAs and tryptophan/serotonin (also known as 5-hydroxytryptamine or 5-HT) are among the best characterized microbial metabolites that are important for MGBA function and are discussed below. 

#### 2.4.1. SCFAs

SCFAs mainly consist of acetate, propionate, and butyrate derived from anaerobic fermentation of dietary fibers by the intestinal microbiota. SCFAs are primarily transported into colonocytes using monocarboxylate transporters (MCTs) or sodium-coupled monocarboxylate transporters (SMCTs) where the majority of SCFAs are metabolized as part of energy production. Non-metabolized SCFAs can be released systemically and may have distant functions in other organs, including the brain [33,34]. Not only serving as colonocyte energy sources, SCFAs generated from microbiota metabolic activity can modulate enteric functions. For example, butyrate and acetate have been shown to protect intestinal barrier integrity via an AMPK-mediated reassembly of tight junction proteins [28,35] and to prevent bacterial internalization and translocation [36]. In addition, SCFAs have also been reported to control the maturation of intestinal immune cells: butyrate produced from *F. prausnitzii* regulated the balance of pro-inflammatory Th17 and anti-inflammatory Treg in IBD [37] by inhibiting the histone deacetylase (HDAC) activity for the expression of *Foxp3*, a critical regulator of T-cell differentiation [38,39]. A failure of intestinal homeostasis regulation by SCFAs could result in both aberrant intestinal inflammation and systemic inflammation.

In addition, SCFAs may distantly influence the brain function by increasing the mobilization of peripheral immune cells or the expression of inflammatory cytokines to modulate systemic inflammation [14]. Alternatively, SCFAs have been shown to activate their receptors (the free fatty-acid receptors, FFAR2 and FFAR3) and associated downstream cAMP-PKA signaling in intestinal EECs, resulting in the release of humoral factors such as GLP-1 and PYY into the circulation [40]. Via both humoral and vagal pathways, these released peptides from EECs can then regulate cognitive function and emotional responses as well as satiety in the brain [41]. Small proportions of SCFAs released into the circulation have been shown to directly enter the brain via the BBB [42] owing to MCT expression in the endothelial cells [33,43]. However, for the regulation of neuroinflammation, it is still controversial whether SCFAs are pro- or anti-inflammatory signals as their influence may be context-dependent [44].

#### 2.4.2. Tryptophan Metabolites and Serotonin

An essential amino acid, tryptophan can be synthesized by several bacteria or provided in food and metabolized to serotonin or kynurenine [32,45]. While serotonin is used as an essential neurotransmitter for multiple biological functions (e.g., mood and cognition) in the brain, it also has crucial roles in the transmission of motor and sensory signals in the intestine. Indeed, approximately 95% of serotonin is produced in the gastrointestinal tract (GIT), indicating its functional dominance in the GIT [46]. The serotonin released into the surrounding regions of the intestine after many types of luminal stimulation regulates GI motility and reflexes via local intrinsic primary afferent neurons and regulates extrinsic primary afferent neurons (including vagal afferents) to affect distant CNS functions such as feeding behaviors [47]. The bioavailability of serotonin must also be tightly regulated using the serotonin reuptake transporter on both nerve endings and in intestinal epithelial cells [48]. 

This enriched serotonin production in the GIT is based on interactions between the microbiota and intestinal cells, as demonstrated by the reduced serotonin levels seen in both plasma and colon samples from GF mice [49]. In fact, serotonin is produced predominantly by enterochromaffin cells (ECs)—a major EEC subtype—in the intestine, and can be activated by the metabolites of spore-forming bacteria [50] and by SCFAs from the gut microbiota [51], concomitant with the increased expression of tryptophan hydrolase 1 (Tph1), a rate-limiting enzyme for 5-HT synthesis in ECs [46,48]. Myenteric interneurons can also produce 5-HT by expressing Tph2 within the enteric nervous system, although this source is quantitatively minor compared to EC production [46,48]. Although direct evidence for 5-HT regulation of intestinal barrier permeability is rare, it has been reported that treatment with 5-hydroxytryptophan (5-HTP), the precursor of 5-HT, regulated the redistribution of the tight junction proteins and sugar permeability in specimens from healthy humans, but not in specimens from IBS patients [52]. In addition, both indole and indole-3-propionic acid (tryptophan derivatives catabolized by specific gut bacteria) were shown to protect gut barrier function [53], suggesting the involvement of tryptophan metabolites in the modulation of intestinal permeability.

Although serotonin can be released directly into the systemic circulation, it cannot penetrate the brain. Instead, it is the availability of BBB-crossing tryptophan and the levels of Tph enzymes in the brain that determine the biosynthesis levels of brain serotonin [54], consistent with a positive correlation between the level of tryptophan in the circulation and that in the hippocampus [55]. Serotonin synthesis can be shunted toward the kynurenine pathway to produce neuroactive kynurenic pathway products such as kynurenic acid and quinolinic acid [45,56], especially when the expression of indoleamine-2,3-dioxygenase 1 (IDO1), the rate-limiting enzyme for tryptophan catabolism, has been increased due to inflammatory cytokines, producing the kynurenic pathway metabolites that can also be served as competitors for regulating the serotonin level [54]. In the brain, kynurenic acid is generally considered to be neuroprotective and quinolinic acid to be excitotoxic, while in the intestine, kynurenic acid is considered to be anti-inflammatory and quinolinic acid to be pro-inflammatory via the regulation of NMDA receptors [57]. However, their exact roles are likely to be dose- and context-dependent and remain to be explored further, especially for microbiota interactions. Interestingly, 5-HT-expressing neurons in anamniotes such as zebrafish are found in several areas of the brain, including the pretectal area, basal forebrain, and hindbrain, but in amniotes they are only found in the hindbrain, although the functional significance of this difference is unclear [54,58].

## 3. MGBA-Associated Neurological Disorders 

Not only does it regulate the normal physiology in the brain, but the gut microbiota is also implicated in pathological conditions of the nervous system via the various gut–brain interactions described above. Acute systemic inflammation due to pathogen infections of the intestine and low-grade chronic inflammation due to intestinal dysbiosis and its derived products of bacterial toxins, metabolites, and cytokines [14] are known to be closely associated with the progression of neurological disorders [59]. Below, the diverse roles of the gut microbiota in autism spectrum disorder (ASD), representing a neurodevelopmental disorder, and AD, representing a neurodegenerative disease, will be discussed in detail. 

### 3.1. Autism. Spectrum Disorder: A Neurodevelopmental Disorder

ASD represents a group of neurodevelopmental disorders often diagnosed in childhood and characterized by impaired social interactions and repetitive/restrictive behaviors that are frequently accompanied by intellectual disabilities and epilepsy [60]. The etiology of ASD is both complex and heterogeneous, as ASD encompasses a range of symptoms with genetic and environmental factors contributing to its pathogenesis. Large-scale exomic and genomic sequencing of ASD patients has identified a number of candidate genes implicated in neuronal connections, synaptic function, chromatin remodeling/transcription, and RNA splicing [61,62,63]. Notably, a gestational inflammatory environment, such as an infection during pregnancy, can dramatically affect neurodevelopmental and autistic outcomes [64]. Based on this idea, inflammatory challenges during pregnancy in animal models, such as viral mimetic poly polyinosinic:polycytidylic acid (I:C) exposure, have been used successfully to establish ASD models for mechanism studies, such as the maternal immune activation mouse model (e.g., [65]).

For the pathobiology of ASD, neurodevelopmental defects in the brain have been the main focus due to both the implication of ASD-susceptible genes in neuronal function and the known morphological abnormalities of ASD brains [66,67]. However, it is also well known that ASD patients have elevated systemic and brain inflammation indicated by increased inflammatory cytokines (e.g., *IL6*, *MCP1*, *IFN1*, and *TNFα*), activated microglia, and autoantibodies [64,68,69]. 

Considering that such abnormal inflammatory regulation is thought to be closely linked to the gut microbiota, dysbiosis has been suggested as a main factor responsible for ASD etiology, and this is also consistent with the comorbid gastrointestinal disturbances of ASD patients and their vulnerability to infections [60,70,71,72]. In support of this idea, GF animals have been reported to exhibit ASD-like traits (e.g., social avoidance and repetitive behaviors) which were rescued by association of the conventional microbiota [73], and both human patients and several ASD mouse models have been shown to have gut dysbiosis [65,74,75,76]. In ASD dysbiosis, altered microbial populations and decreased diversity have both been documented, with *Clostridium* spp. proposed to be the main culprit by producing neurotoxins that are transported via the vagus nerve (e.g., [77]), but consistent changes in microbiota profiles have yet to be identified [75]. The most direct evidence for a causal relationship between the gut microbiota and ASD has come from recent “humanized” ASD mouse models: the offspring from GF mice harboring a gut microbiota from ASD-patients exhibited ASD-like phenotypes, and further transcriptomic analyses identified aberrant alternative splicing of ASD-risk genes similar to that seen in human ASD brains [78]. In support of a microbiota role (and their metabolites) for such ASD-related phenotypes, ASD model mice were rescued by a variety of approaches, including the use of prebiotics/probiotics (*Lactobacillus reuteri*), antibiotics (vancomycin), metabolites (GABA agonists taurine and 5-aminovaleric acid), specific diets (a ketogenic diet), and microbial transfer therapy [74,78,79,80].

The etiology of ASD, especially as it relates to GI problems, is postulated to begin with increased gut permeability (“leaky gut”) in the offspring due to gut dysbiosis under conditions of elevated inflammation such as during maternal immune activation (MIA) or with a high-fat diet (HFD) [8,65,81]. An increased level of toxic metabolites, such as 4-ethylphenylsulfate (4-EHP) and 5-HT [65,82], or a decreased level of beneficial metabolites, such as butyrate during dysbiosis [28], may also impact gut permeability by modulating the expression of tight junction proteins in the intestine [75]. Such a dysfunctional intestinal permeability may allow for the unrestricted entry of dietary components, bacterial components, and metabolites that can elicit increased local intestinal inflammation that is coupled with increased plasma levels of pro-inflammatory cytokines (e.g., IL-6), resulting in systemic inflammation [14] or the permeability changes may directly disturb vagus nerve-dependent signaling to the brain [17]. Notably, IL-6 has also been shown to impair tight junctions by inducing pore-forming claudin-2 [83], further worsening gut permeability. It is well established that systemic inflammation can disrupt BBB integrity in neurological disorders [10]. As a result, the compromised BBB may be permissive to peripheral pro-inflammatory cytokines (e.g., IL-6), metabolites (e.g., p-cresol and propionate), or the infiltration of peripheral immune cells in ASD patients [84,85], with ensuing increased neuroinflammation. Dysregulated neuroinflammation in ASD is known to play a crucial role in its pathogenesis and is mediated by activated microglia and astrocytes that impact dendritic branching, spine density, and neuronal connectivity as well as elevated cytokine expression, finally leading to both cognitive and behavioral abnormalities [86,87].

Therefore, treating ASD by gut microbiota manipulation may offer more efficient therapeutic approaches which include antibiotics, prebiotics, probiotics, beneficial metabolites, a gluten-free diet, or fecal microbiota transplantation. For example, butyric acid administration is known to ameliorate ASD phenotypes in both mouse models and human patients, presumably by modulating mitochondrial function and neurotransmitter gene expression; interestingly, other SCFAs (e.g., acetate and propionate) did not show any rescue effect; rather, propionate has been used to reversibly induce ASD phenotypes and to generate ASD animal models [75]. Furthermore, in several ASD models where mice exhibited gut dysbiosis (e.g., maternal HFD-fed mice, BTBR mice, and *shank3* knockout mice) supplementation with *L. reuteri* was able to rescue the ASD-like social interactions, although this was achieved by vagus nerve stimulation that activated oxytocin release from specific neurons in the ventral tegmental area of the brain, and not by microbial changes [74,88]. Both gluten-free and ketone-rich diets have been reported to rescue GI abnormalities and ASD symptoms, probably by regulating mitochondria-associated energy metabolism and oxidative stress [89] as well as by modulating the gut microbiota [90,91]. In addition, direct “microbial transfer therapy”, using two weeks of vancomycin treatment followed by eight weeks of fecal microbiota transplantation, has been shown to improve these gut abnormalities and autistic phenotypes and to change the abundance of beneficial bacteria in ASD patients [92]. However, as these interventions may often have unwanted side effects such as constipation, and sometimes have conflicting therapeutic effects, more detailed mechanistic studies and optimization protocols are still required for personalized applications.

### 3.2. Alzheimer’s Disease: A Neurodegeneration Problem

There are two forms of AD: early onset (EOAD, or familial) and late onset (LOAD), with the latter accounting for more than 95% of cases that usually occur after the age of 65. AD is pathologically characterized by the accumulation of amyloid β (Aβ) species and phosphorylated tau protein, leading to the formation of neurotoxic amyloid plaques and neurofibrillary tangles in the brain, respectively, in addition to neuroinflammation, loss of synapses, cognitive impairment, and the eventual loss of neurons [93,94]. The non-genetic risk factors that are well known for AD include aging, some metabolic disorders, and diets [95,96,97], and both large-scale genome-wide associated studies (GWAS) and whole-genome sequencing of AD patients have identified more than 30 high-risk genetic loci for the disease [98]. 

Previous studies have suggested that these risk factors—aging along with some metabolic syndromes and diets—may contribute to AD pathogenesis through interactions with the gut microbiota. It has been well established that these factors are tightly linked to changes in the microbiome [99,100,101]. In addition, the host’s *APOE* alleles (especially *APOE4*), representing the strongest AD genetic risk factor, have been correlated with both an elevated innate immune response and changes in the butyrate-producing bacterial populations and their metabolite profiles in human and mouse models [102,103]. Similarly, several genetic risk genes for AD, including *TREM2*, *CD33* (also known as *SIGLEC-3*), and *SHIP1* have also been implicated in intestinal inflammation [104,105,106], which can directly or indirectly interact with the gut microbiota. The close relationship between gut dysbiosis, systemic inflammation, and AD pathogenesis has been shown in a clinical study where increases or decreases in the bacterial populations harboring either pro-inflammatory (e.g., *Escherichia/Shigella*) or anti-inflammatory (e.g., *Eubacterium rectale*) activities, respectively, were associated with systemic inflammation status and brain–amyloid deposition in cognitively impaired patients [107]. Similarly, a population study of IBD patients revealed that increased intestinal inflammation (likely coinciding with gut dysbiosis) was associated with both a higher early onset risk of AD and a higher overall incidence of AD [101]. Numerous correlational studies of gut dysbiosis in AD patients and in AD mouse models (e.g., APP/PS1, 5XFAD, and P301L transgenic mice) have extensively characterized changes in microbial populations, and these results have been summarized in recent reviews [59,108,109]. The importance of the gut microbiota as a contributing factor to AD has been functionally validated using GF and antibiotic-treated transgenic AD mouse models that exhibited reduced AD-like symptoms [110,111].

In the multifactorial etiology of LOAD, brain inflammation, proposed as one of the most crucial processes for its development [112], is known to be closely associated with microbiota and permeability changes in the gut, as described above. In neurological disorders, the GIT barrier has been found to be defective due to gut dysbiosis and altered microbial metabolites such as SCFAs and tryptophan metabolites [14], and the integrity of the BBB has also been shown to be compromised during the early stages of AD, irrespective of amyloid β and tau accumulations [113,114,115]. Neuroinflammation-related activated microglia and the increased expression of pro-inflammatory cytokines in the brain may both originate from such permeability defects in the GITs and brains of AD patients. Such defects may also allow the direct entry of pro-inflammatory bacteria such as oral pathogens (e.g., *Porphyromonas gingivalis*) and their endotoxic components/metabolites into the brain [116,117]. For example, infiltrated lipopolysaccharide (LPS), a representative microbe-associated molecular pattern molecule (MAMP), potentially originated from those pathogens, has been shown to promote Aβ production and aggregation as well as neuroinflammation [118,119] or to indirectly activate cells lining these barriers and the peripheral nervous system to elicit systemic inflammation and facilitate immune cell infiltration into the brain and inflammatory cytokines [120].

Interestingly, specific microbiota members in humans can also produce bacterial amyloid proteins, such as Curli produced from the *csgA* gene in *E. coli*. These bacterial amyloid proteins can prime amyloidosis and brain proteinopathies in neurodegenerative diseases through cross-seeding activity to form β-sheet structure-mediated toxic aggregates in a “prion-like” fashion via the autonomic nervous system, or by eliciting gut inflammation and releasing inflammatory mediators [121]. Although interactions between Curli and amyloid β of AD have yet to be tested directly, it has recently been demonstrated that Curli promoted the aggregation and pathology of α-synuclein, the protein responsible for Parkinson’s disease progression in vitro and in vivo [122].

## 4. Zebrafish as a Model System for MGBA Studies

**Figure 2:**
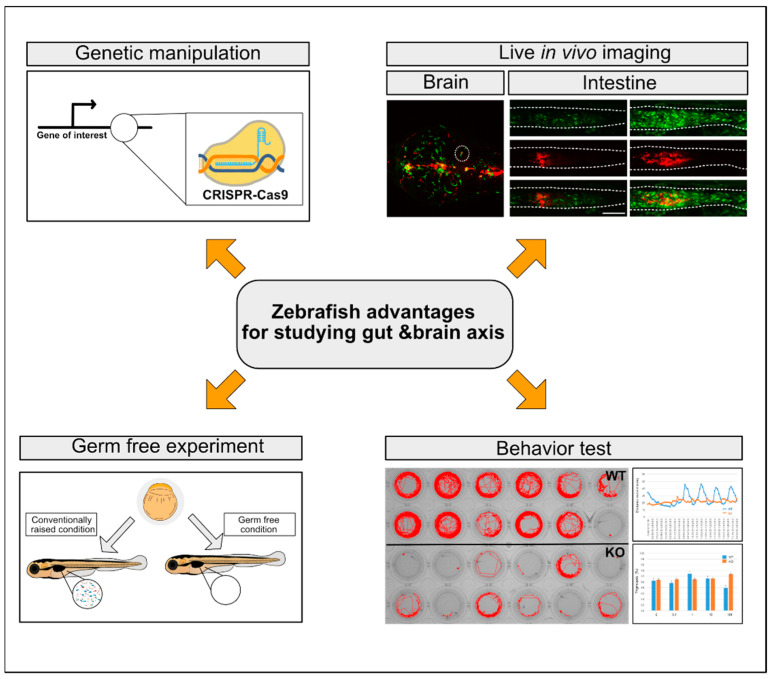
Key advantages of the zebrafish model for studying the MGBA. Refer to the text for details.

The zebrafish model became popular for genetic studies due to its large clutch size, small body size, genomic and physiological similarities to humans, its body transparency optimal for in vivo imaging, powerful forward and reverse genetics, and the possibility of in vivo chemical screening. These advantages allowed the zebrafish to become the main model organism for studying developmental, physiological, and pathological processes, and as a model with a large collection of mutants and transgenic animals that can visualize both tissues of interest and signaling pathways, it can mimic a variety of human disease conditions for understanding their underlying mechanisms [123]. Although the molecular components and signaling pathways responsible for MGBA functionality (i.e., those of dysbiosis, dysfunctional immune cell activities, and aberrant metabolites) have been identified mainly from mouse model studies, the zebrafish model has emerged as an alternative and attractive vertebrate model with its advantages of being amenable to genetic manipulations, allowing for real-time in vivo imaging capability at the whole organism level, and the availability of powerful and easy GF-rearing, gnotobiotic conditions. Indeed, host gut microbiota interactions [124] and neurological disorders [125,126] that mirror human conditions have been independently investigated using zebrafish. However, the combined efforts of the characterization of recently developed zebrafish disease models for ASD and AD, the establishment of microbial approaches including GF culture [127,128] and metagenomic analyses of the microbiota [129,130], and the expansion of their neurobehavioral repertoires [131,132,133,134] may allow dissection of the signaling pathways for the host–bacteria interactions in neurological disorders, discussed in the following sections. The key advantages of the zebrafish model for studying the MGBA are summarized in Figure 2.

### 4.1. The Zebrafish GIT and Associated Cell Types

Both the development and the organization of the zebrafish intestine have been well documented in previous studies: although the zebrafish GIT lacks an acidic stomach, crypts, Paneth cells, and classical microfold (M) cells, the anatomical, molecular, and functional features of its GIT are largely conserved between mammals and zebrafish [135,136,137]. The major cell types identified in the zebrafish GIT include absorptive enterocytes, mucin-secreting goblet cells, hormone-releasing EECs, immune cells, smooth muscle cells, and an enteric nerve system [135,138]. These cell types in the zebrafish GIT play pivotal and distinct roles in coping with diverse environmental changes and can relay this information to other organs, including the brain. For example, their EECs are capable of sensing luminal contents while still producing and releasing hormones or signaling molecules that can regulate physiological and homeostatic functions of the intestine and the brain [139]. These EECs can be dysfunctional, with morphological alterations after HFD feeding in a larval model [140], and can secrete 5-HT in response to a pathogen infection (*Edwardsiella tarda*) to activate enteric neurons and promote bacterial clearance mediated by the Trpa1 receptor [141]. 

A zebrafish vagus nerve has also been described in both embryos and adults, forming at approximately 3–4 dpf [142], and an afferent sensory circuit of the vagus (projecting to the hindbrain via the nodose ganglion) was elegantly shown to be activated by both an *E. Tarda* infection or its tryptophan metabolites [141]. The zebrafish enteric nervous system (ENS), a neural crest-derived peripheral nervous system, consists of enteric neurons, a submucosal/myenteric plexus, associated glia, and muscle layers. It has been shown to regulate intestinal motility and to mediate connectivity between the CNS and the intestine, with neurons secreting neurotransmitters such as serotonin, dopamine, histamine, acetylcholine, and GABA, similar to mammals [143]. The developmental programs of the zebrafish ENS have been well described using forward and reverse genetic studies, revealing the intricate signaling pathways governing ENS genesis and function in both normal and pathological contexts that are relevant to human conditions [144,145]. In addition, the zebrafish immune system has shown a high level of homology to that of mammals: most immune cell lineages in mammals (e.g., macrophages, neutrophils, and lymphocyte B/T cells) have also been identified in zebrafish [146] and many mammalian immune-signaling pathways, immune receptors (pattern recognition receptors such as TLRs), and inflammatory mediators such as cytokines, interleukins, and complement are conserved in zebrafish [147].

### 4.2. Genetic Approaches to Loss-of-Function and Gain-of-Function Studies with Zebrafish Transgenic Lines

#### 4.2.1. Loss-of-Function Approaches

For both efficiency and convenience, morpholino oligonucleotides have been commonly used in zebrafish to transiently knock down the endogenous expressions of genes of interest in loss-of-function studies [148]. These morpholinos can be designed to disturb translation by binding to translation initiation sites, or to disturb RNA splicing by binding to splicing sites, which affects zygotic or maternal zygotic expression of the mRNA, respectively. Although a downside of morpholino administration is potential toxicity which can give rise to unanticipated phenotypes and misinterpretations of the gene functions [149], the technique can still be a useful loss-of-function genetic tool to quickly test gene function as long as one carefully follows the guidelines for proper usage [150].

Permanent loss-of-function studies require zebrafish lines with mutant knockouts, and the zebrafish model represents an ideal system to apply recent genome-editing technology. Traditionally, collections of zebrafish mutant lines with diverse phenotypes were obtained by forward genetic screening using random mutagenesis, with large-scale mutagenesis screening generating approximately 1500 mutant lines [151]. The generation of gene-targeted zebrafish knockouts using reverse genetic approaches became available and popular when genome-editing technologies were adopted, beginning with zinc finger nucleases (ZFNs), transcription activator-like effector nucleases (TALENs), and most recently, clustered regularly interspaced short palindromic repeats (CRISPR) editing which was originally identified as a bacterial defense system against phage infection and was adapted to allow rapid and accurate target gene editing in vivo [152]. Ever since the CRISPR/Cas9 system was successfully validated to produce zebrafish knockouts in vivo [153], the technique has transformed zebrafish reverse-genetic approaches to allow for the generation of mutants at will, and has also been applied to the introduction of knockin mutations, the target-specific transcriptional regulation, and induction of precise base-pair editing [154,155].

#### 4.2.2. Gain-of-Function Approaches

Gain-of-function studies usually require the overexpression of genes of interest. Transient overexpression can be achieved by the direct in vitro microinjection of transcribed mRNA encoding the protein of interest into one-cell zebrafish embryos. Although this is not cell type-specific overexpression, mRNA-injected overexpression is a fast and valuable tool for functional gene analysis, and also can be used to verify a loss-of-function phenotype in rescue experiments (e.g., [156]). Both long-term and tissue-specific gene expression effects require permanent/stable expression in transgenic animals with cell type or tissue specificity. Such stable transgenic animals, with germline transmission, can be created with high efficiency by microinjecting plasmid DNA made using swappable promoters, the genes of interest, and effectors (e.g., fluorescence proteins and recombination cassettes) using a transposon-based Tol2 kit [157] which can direct gene expression in a tissue-specific manner. To make the use of cell type-specific expression even more flexible, the Gal4/UAS binary system has been adopted because it permits any gene to be expressed in a desired place and time by crossing tissue-specific Gal4 transgenic lines with a UAS gene of interest [158]. However, Gal4 cytotoxicity and UAS methylation have hampered any broader application of the Gal4/UAS system in zebrafish [159]. Attempts to circumvent such GAL4/UAS system problems, such as fusing the Gal4 DNA-binding domain to heterologous transactivation domains, and optimizing the 5X UAS sequence [160], or the use of alternative flexible binary systems such as the Cre/loxP system, the Flp/FRT system, and the QF/QUAS system have also been developed and are still evolving in the zebrafish research field [161,162,163].

#### 4.2.3. Zebrafish Transgenic Lines and In Vivo Imaging of Host-Bacterial Interactions

Due to the optical transparency of the zebrafish embryo throughout its development, and the capability for real-time in vivo imaging at high resolution, reporter transgenic lines that can visualize immune cells and immune signaling will become great assets for dissecting interactions between host immune cells and bacteria, in addition to dissecting gut–brain signaling. Several fluorescently tagged transgenic lines have been established that have labeled myeloid cells (neutrophils, macrophages, and microglia), lymphocytes (B cells and T cells), and intestinal cells (enterocytes and EECs) that are summarized in Table 1 and Table 2. The use of these same cell type-specific promoters can also drive the expression of a variety of advanced genetically encoded fluorescent proteins (e.g., photoconvertible Kaede or Dendra) and genetically encoded neurotransmitter sensors (e.g., dopamine and norepinephrine) that have been developed by coupling neurochemical-sensing G-protein-coupled receptors (GPCRs) with a circular-permutated fluorescent protein (Table 3). Such transgenic zebrafish lines have been suitable for in vivo cell tracking to reveal direct communications between the gut microbiota and the brain by UV photoconversion of Kaede in a non-invasive manner [164] and for the in vivo monitoring of dynamic zebrafish larval brain responses to dopamine stimulation in real time [165]. In addition, a tissue-specific ablation strategy, where *nfsB* encoding nitroreductase B (NTR) converts prodrugs (e.g., metronidazole) into cytotoxic metabolites and ablates cells expressing NTR, can also be applied to explore the functional roles of specific gut-derived cell types during brain development [164]. Therefore, the exploitation of these powerful genetic approaches combined with a variety of in vivo imaging techniques for monitoring both cellular behavior and signaling pathways in whole zebrafish may reveal novel mechanistic insights into microbiota–gut–brain interactions, especially in the context of neurological disorders such as ASD and AD.

### 4.3. The Gut Microbiota of Zebrafish in MGBA Studies

In zebrafish, intestinal colonization by microbes from the surrounding environment begins at approximately 3–4 dpf as the mouth opens and the GIT matures [129]. The core microbiota indigenous to the zebrafish GIT, dominated by phyla Proteobacteria and Fusobacteria, have been shown to be determined by selective pressure from the zebrafish host based on comparative composition analyses of the gut microbiota in mouse–zebrafish reciprocal transplantation, domesticated strains, and wild-caught zebrafish [185,186]. Although the exact taxonomies of the microbial communities in humans, mice, and zebrafish differ, there is a trend towards interspecies conservation at the phylum level [187,188]. In addition, host responses to gut colonization by microbiota have been reported to be significantly shared between zebrafish and mouse models [189], and zebrafish, mouse, and human microbiomes are known to have similar abundances of functional pathways (i.e., DEG pathways) [130] which suggests that functional and mechanistic findings using the zebrafish microbiota may have direct translational implications for understanding human microbiota functions.

Roles for gut microbiota in GIT development and function have been investigated using several zebrafish models, and our mechanistic understanding of host gut microbiota interactions has benefited from using both GF and microbial reassociation approaches. The protocol for rearing zebrafish larvae under GF conditions for up to 8–9 dpf has been well established [127,189,190]. Although gross morphology was normal, GF zebrafish larvae exhibited defective proliferation and differentiation of intestinal epithelial cells (e.g., depletion of goblet cells and EECs, immature glycan expression, and a lack of intestinal alkaline phosphatase activity at the brush border) which could be rescued by reassociation with gut microbes [189,190]. It was also found that the residential gut microbiota decreased their own potential toxicity by inducing the expression of the intestinal alkaline phosphatase (*iap*) gene localized in the brush border of the intestine to detoxify LPS, and because GF zebrafish larvae failed to induce *iap*, they were rendered hypersensitive to LPS [191]. The gut microbiota can also contribute to the functional regulation of the immune system, as shown by the NFkB-dependent and tissue-specific immune-signaling changes seen after microbial colonization compared to GF controls that were visualized in vivo in real-time using *Tg(NFkB:EGFP)* transgenic zebrafish larvae [181] and by the dramatic induction of the intestinal serum amyloid A (*saa*) gene upon microbial colonization that contributed to innate immunity by suppressing the aberrant activation of neutrophils [178].

Novel mechanistic findings about how the microbiota can regulate host metabolism have also been made using zebrafish models. For example, metabolically, the presence of the microbiota promoted fat uptake and the formation of lipid droplets upon feeding that was concomitant with phylum Firmicutes enrichment [192] and “silenced” EECs by damaging their nutrient-sensing ability under HFD conditions, concomitant with the genus *Acinetobacter* blooming [140]. In addition, probiotic *Lactobacillus* treatment modulated the gut microbiota composition and the gene expression signature of glucose metabolism in 8 dpf zebrafish larvae, resulting in both reduced glucose levels and feeding behaviors, presumably through the increased formation of SCFAs by gut microbiota activity [193]. For modelling type 2 diabetes in adult zebrafish, changes in both microbial compositions and gene expression profiles towards more diabetes-related patterns have also been reported, consistent with mammalian models [194]. For toxicology research, the GF zebrafish model has been used to address interactions between the gut microbiota and the metabolism of xenobiotics such as chemical drugs and toxic pollutants [195], which has broadened the applicability of zebrafish microbial studies in combination with the power of both GF and reassociation experimental designs.

Similar to GF mice exhibiting increased locomotion, decreased anxiety, impaired memory, and decreased social behavior in neurodevelopmental and neurobehavioral studies [7,196], GF-reared or antibiotic-treated zebrafish larvae have consistently shown hyperactivity and anxiolytic activity based on locomotion and thigmotaxis assays, respectively, which could be rescued by their exposure to commensal microbiota (“conventionalization”) or to specific bacteria [197,198]. In addition, supplementing larval and adult zebrafish with *Lactobacillus* species demonstrated that these probiotic bacteria conferred anxiolytic effects and increased shoaling, and these behavioral effects were accompanied by changes in both microbial composition and gene expression profiles in the brain related to neurotransmitter signaling and Brain-Derived Neurotrophic Factor (BDNF) [199,200], suggesting this zebrafish model as a platform for validating the potential use of probiotics for neurobehavioral effects.

### 4.4. Zebrafish Models for the Neurological Diseases ASD and AD

#### 4.4.1. The Zebrafish ASD Model Representing a Neurodevelopmental Disorder

Zebrafish models for ASD have been well established using chemical treatments or genetic manipulations followed by neurobehavioral assays to assess a range of core autism-like phenotypes, including disturbances of social behavior, inhibitory avoidance, aggression, anxiety, and repetitive behaviors [201,202]. For chemical treatments, the glutamatergic N-methyl-D-aspartate (NMDA) receptor antagonist MK-801, the dopamine D1 receptor antagonist SCH23390, and the epilepsy and bipolar disorder drug valproic acid have been used to evoke ASD-like symptoms in rodents [203] and have also been applied to zebrafish for modeling ASD [204,205]. For genetic modeling analogous to rodent ASD models which were established by overexpressing ASD-risk genes such as *kctd13*, *bbs7*, and *cep290*, or by knocking down/knocking out *dyrk1a*, *coro1a*, *fam57ba*, *gdpd3*, *hirip3*, *kif22*, *maz*, *ppp4ca*, *shank3*, *fmr1*, and *cntnap2*, zebrafish ASD models have been successfully generated by targeting a similar set of ASD-susceptibility genes using genetic manipulations (reviewed in [126,206]). 

As an example, the zebrafish *dyrk1aa* knockout mutant (an ASD-causative gene also implicated in Down syndrome) exhibited a smaller brain size with increased cell death, abnormal brain activity, and social interaction deficits reminiscent of typical ASD symptoms [207]. The *shank3b* knockout mutant, another zebrafish ASD model, also showed impaired and repetitive locomotion behavior and social interaction defects, together with decreased expressions of synaptic proteins [208], while shank3a/b double knockouts exhibited gastrointestinal motility defects with reduction of serotonin-positive EECs [209]. However, despite extensive experimental evidence in both human and mouse models supporting the involvement of microbial composition dysbiosis in ASD pathogenesis (reviewed in [78,210], zebrafish studies on the role of the gut microbiota underpinning ASD pathogenesis are surprisingly lacking, except for a very recent report that tested the requirement of early microbial colonization for acquiring social interaction behaviors: zebrafish reared under GF conditions, followed by colonization at 7 dpf, exhibited social behavior defects as a result of aberrant axonal arbor complexity in the ventral nucleus of the ventralis telencephali (Vv) and neuronal connectivity defects. These defects were also accompanied by a decreased abundance of microglia in the forebrain, highlighting a key role for microglia in the mediation of microbiota signaling and neuronal development [211].

#### 4.4.2. The Zebrafish AD Model Representing a Neurodegenerative Disease

Similar to their use in rodent models of AD, the accumulation of Aβ42 peptides and the aggregation of phosphorylated tau protein have been employed to generate zebrafish AD models either by direct Aβ42 peptide injections into the brain or by the overexpression of amyloid β42 or a phosphorylation-prone mutant form of tau protein to create stable transgenic zebrafish. Initial studies showed that direct injections of Aβ42 into the hindbrain ventricle induced cognitive deficits and tau phosphorylation which could be rescued by lithium, a GSK3β inhibitor [212]. Since then, research has shown that Aβ42 injections into the brain ventricle during both larval and adult stages have validated the chaperone activity of gold nanoparticles to mitigate Aβ toxicity using a larval locomotion assay and an avoidance test [213] and has also revealed Aβ42 molecular pathways regulating the sleep/awake cycle [214]. By taking advantage of the regenerative capacity of the adult zebrafish brain after Aβ42 brain injection, IL4 was identified as an activator of neural stem cell proliferation for regeneration after Aβ-induced neuronal death in the adult brain by suppressing serotonin levels to disinhibit the BDNF–NFkB signaling axis for regeneration [215,216]. As the tryptophan metabolic pathway is well conserved in teleosts, including zebrafish [54], zebrafish models may be useful tools to better understand the contributions of tryptophan metabolism to brain tissue regeneration in neurological diseases at a systemic level and in the context of the MGBA axis.

Stable zebrafish AD transgenic lines have been generated mostly to model tauopathy, with neuron-specific overexpression of human *TAU* mutations. Among these, a representative zebrafish tauopathy model overexpressed human *TAU* with a P301L mutation in the nervous system using the Gal4–UAS system [217]. This model recapitulated tau pathology, with aberrant phosphorylation, neurofibrillary tangle formation, increased neuronal cell death, and locomotion defects. Furthermore, the candidacies of GSK3β inhibitors were successfully validated using this model by assessing the amelioration of tau phenotypes with candidate treatments [217]. Another zebrafish tauopathy model, using the overexpression of *TAU* with a rare mutation (A127T), exhibited defective motor axons and proteasome dysfunction concomitant with insoluble tau protein accumulation that could be rescued by activating autophagy [218]. It is noteworthy that even with identical mutations, *TAU* expressions do not always induce tauopathy phenotypes, even with tau protein phosphorylation as reported by [219]. Importantly, given the central importance of AD within the scope of neurodegenerative diseases, and the long history of using several model organisms in AD research, the wide uses of zebrafish AD and tauopathy models are still to be established and further improved in order to recapitulate human AD pathology more precisely. These factors currently hamper the application of zebrafish AD models to MGBA studies.

## 5. Future Directions

Over the last few decades our understanding of the interactions between the microbiota, the gastrointestinal tract, and the brain that can affect both the physiology and pathology of neurodevelopmental and neurodegenerative conditions has been significantly broadened, with rodents predominantly being used as animal models. As a model organism, the zebrafish is beginning to be used for MGBA studies, based on their molecular, genetic, and neurobehavioral advantages and the ability of zebrafish models to recapitulate neurological disorders. One could imagine the development of a platform for “humanized” zebrafish ASD models, where gut microbiomes from human ASD patients would be exposed to an array of GF-reared knockout and knockin zebrafish and a high-throughput chemical screening would be performed based on ASD-relevant phenotypes for personalized medicine targeting. By taking advantage of improved genetic zebrafish models that can better recapitulate the diverse neurological symptoms of human patients with faster, more reliable, and convenient assays, we expect that the combination of a high-resolution in vivo imaging at the whole animal level and established microbial manipulations will lead to novel mechanistic insights into the neuronal circuits and MGBA components in the near future that will lead to a better understanding of neurological disorders and innovative therapeutic approaches.

## Figures and Tables

**Table 1:** Zebrafish transgenic reporter lines labeling various immune cell types.

Cell Type	Promoter (Gene Name)	Zebrafish Transgenic Line	Features	References
Myeloid cell	*spi1b/pu.1 (Spi-1 proto-oncogene b)*	*Tg(spi1b:GAL4,UAS:EGFP)*	*GFP* or *RFP* expression combined with other UAS lines	[166]
		*Tg(spi1b:GAL4,UAS:TagRFP)*		
Neutrophil	*mpx (myeloid-specific peroxidase)*	*Tg(mpx:GFP)*	*GFP* or *mCherry* expression	[167]
		*Tg(mpx:mCherry)*		[168]
	*lyz (lysozyme)*	*Tg(lyz:EGFP)*	*GFP* expression	[169]
Macrophage	*mpeg1.1 (macrophage expressed 1, tandem duplicate 1)*	*Tg(mpeg1:dendra2)*	*Dendra2* converted by UV from green to red for cell tracking	[170]
		*Tg(mpeg1: GFP-CAAX)*	Expression of *membrane-GFP* in macrophages	[166]
	*mfap (microfibril associated protein 4)*	*Tg(mfap4:dLanYFP-CAAX)*	Expression of *membrane-YFP* in macrophages	[170]
	*csf1ra/fms (colony stimulating factor 1* *receptor, a)*	*TgBAC(csf1ra:GFP)*	*GFP* expression	[171]
		*Tg(fms::nfsB-mCherry)*	*mCherry* expression conditional cell ablation by metronidazole treatment	[172]
	*irg1 (immunoresponsive gene 1)*	*Tg(irg1:EGFP)*	*GFP* expression Expression of activated macrophages	[173]
Microglia (the resident innate immune cells in the brain)	*apoeb (apolipoprotein Eb)*	*Tg(apoeb:lyn-EGFP)*	expression of *membrane-GFP* in microglia	[174]
	*slc7a7 (solute carrier family 7 member 7)*	*Tg(slc7a7:Kaede)*	Microglia precursor expression *Kaede* converted by UV from green to red for cell tracking	[172]
	*p2ry12 (purinergic receptor P2Y12)*	*TgBAC(p2ry12:p2ry12-GFP)*	*GFP* expression	[175]
T cell	*lck (LCK proto-oncogene, Src family* *tyrosine kinase)*	*Tg(lck:GFP)*	*GFP* expression	[171]
B cell	*ighm* *(immunoglobulin heavy constant mu)*	*Tg(IgM1:eGFP)*	*GFP* expression	[176]
nodose ganglia (vagus nerve)	*isl1 (ISL LIM Homeobox 1)*	*Tg(isl1:EGFP)*	a subset of *isl1*+ cells	[141]
enterocyte	*cldn15la (claudin 15-like a)*	*TgBAC(cldn15la-GFP);* *Tg(-0.349cldn15la:* *mCherry)*	absorption	[177,178]
enteroendocrine	*neurod1 (Neuronal Differentiation 1)*	*TgBAC(neurod1:EGFP)* *Tg(neurod1:RFP* *)*	intestinal hormone release	[140]
	*nkx2.2a (NK2 homeobox 2a)*	*Tg(nkx2.2a:mEGFP)*	intestinal hormone release	[179]

**Table 2:** Zebrafish transgenic reporter lines to monitor the spatial and temporal activities of immune-signaling components.

Immune-Signaling Component	Description	Zebrafish Transgenic Line	References
*myd88* *(myeloid differentiation response gene 88)*	major adaptor protein of Toll-like receptor (TLR) –mediated signaling	*Tg(myd88:EGFP)* *Tg(myd88:DsRED2)*	[180]
*NF-κB* *(nuclear factor κ-light chain enhancer of activated B cells)*	transcription factor; activated by bacteria-derived cytokines	*Tg(NF-κB:EGFP)*	[181]
*il1β* *(interleukin 1, beta)*	pro-inflammatory cytokine	*TgBAC(il1β:eGFP)*	[182]
*tnf-α* *(tumor necrosis factor α)*	pro-inflammatory cytokine	*Tg(tnf-α:GFP)*	[183]

**Table 3:** Zebrafish transgenic reporter lines for neurotransmitter sensors and genetically modified fluorescent proteins.

MGBA Cell Types	Description	Zebrafish Transgenic Line	References
dopamine	in vivo dopamine sensor	*Tg(elval3: DA1m)*	[165]
norepinephrine	in vivo norepinephrine sensor	*Tg(HuC:GRAB_NE1m_)*	[184]
Kaede	UV-photoconvertible sensor	*Tg(Gal4-VP16;UAS:EGFP)* X *Tg(UAS:Kaede)*	[164]
CaMPARI	UV-photoconvertible calcium sensor	*Tg(neurod1:CaMPARI)*	[141]

## Data Availability

Not applicable.

## Abbreviations

5-HT	5-hydroxytryptamine
AD	Alzheimer’s disease
Aβ42	amyloid β42
ASD	autism spectrum disorder
BBB	blood–brain barrier
CNS	central nervous system
CSF	cerebrospinal fluid
EECs	enteroendocrine cells
GF	germ-free
GIT	gastrointestinal tract
GABA	gamma-aminobutyric acid
IBD	inflammatory bowel disease
IFNγ	interferon γ
LPS	lipopolysaccharides
MAMP	microbe-associated molecular pattern
MGBA	microbiota–gut–brain Axis
SCFAs	short chain fatty acids
TNF	tumor necrosis factor
